# Application of *Pistacia atlantica* Pickering emulsion‐filled chitosan gel for targeted delivery of curcumin

**DOI:** 10.1002/fsn3.3962

**Published:** 2024-01-16

**Authors:** Sara Naji‐Tabasi, Monir‐sadat Shakeri, Atena Modiri‐Dovom, Saeedeh Shahbazizadeh

**Affiliations:** ^1^ Department of Food Nanotechnology Research Institute of Food Science and Technology (RIFST) Mashhad Iran; ^2^ Department of Food Biotechnology Research Institute of Food Science and Technology (RIFST) Mashhad Iran

**Keywords:** Baneh gum, curcumin, hydrogel, Pickering emulsion

## Abstract

Emulsion‐filled hydrogels are a growing system in the food industry for delivering bioactive compounds. In this study, Baneh gum (BG) particles were prepared as a Pickering emulsion stabilizer for curcumin delivery. Then, BG Pickering emulsion was added to the chitosan solution (1.5%, 2.0%, and 2.5% w/w) in different Pickering emulsion (PE):hydrogel (HYD) ratios (1:3, 1:5, and 1:7) to create an emulsion‐filled gel. The highest amount of Cur stability after the 3rd week of storage was observed in the sample containing 2.0% CS and a 1:7 PE:HYD ratio (97.36%). Pickering emulsion and emulsion‐filled gel significantly protected the antioxidant activity of curcumin against the thermal process (*p* < .05). Curcumin loading in the emulsion‐filled gel provided better protection against the gastric condition compared to the emulsion system. The chitosan hydrogel swells in an acidic environment, but its combination with the anionic structure of the emulsion causes a lower release of curcumin in the stomach environment, which can help the stability of curcumin in the digestive system and have a controlled release in the gastrointestinal tract.

## Graphical abstract

Pickering emulsion and emulsion‐filled gel significantly protected the antioxidant activity of curcumin against the thermal process. Curcumin loading in the emulsion‐filled gel provided better protection against the gastric condition compared to the emulsion system.

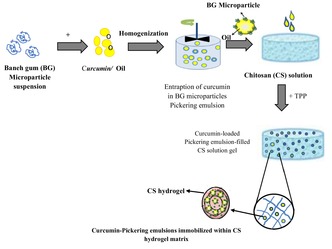

## 1 INTRODUCTION

Recently, Pickering emulsions (PE) have attracted extensive interest due to their advantages of being “surfactant‐free” and sustained delivery of bioactive compounds (Dai et al., 2018). Edible Pickering particles‐stabilized emulsion is expected to act as a promising system for the protection, encapsulation, and oral delivery of bioactive compounds (Xiao et al., 2017). Nano‐encapsulation of bioactive compounds by Pickering emulsions could protect their properties (antioxidant, stability, and solubility, etc.) (Liu et al., 2018). A dense shell of solid particles is formed around the Pickering emulsion droplets, which acts as a barrier, and in some cases, the polymers of the inner layer can interact with the loaded bio‐compounds. Thus, a better‐sustained release happens. Yang et al. (2017) reported that Pickering emulsion is the perfect carrier for controlling the delivery of bioactive compounds (Yang et al., 2017).

Although Pickering emulsions exhibit high stability against coalescence and have protective effects on the lipid phase during storage, they cannot withstand high temperatures and alkaline treatments. Therefore, the structural integrity of emulsion droplets is destroyed because of proteolysis in gastric digestion fluid (Xiao et al., 2017). Designing an oral delivery system based on Pickering emulsions with increased resistance to gastric conditions and harsh processing to increase the stability of bioactive compounds in the gastrointestinal tract is one of the main challenges for edible particles‐stabilized Pickering emulsions (Xiao et al., 2017). To overcome the limitation of the responsiveness of particle‐based Pickering stabilizers to gastrointestinal digestion, it is necessary to develop new systems to integrate the valuable properties of Pickering emulsion and bulk hydrogels (Farjami & Madadlou, 2017; Meid et al., 2011; Zhao et al., 2015). The objective of this study was to stabilize curcumin‐loaded Pickering emulsion (cur‐PE) within the hydrogel matrix to develop sequential release properties.

The hydrogels are three‐dimensional, cross‐linked polymer networks. A common feature of hydrogels is that their structure remains stable under temperature and pH changes. Stimuli‐sensitive hydrogels can be divided into several types, including biologically sensitive (enzymes), chemically sensitive (glucose and pH), and physically sensitive (electricity, temperature, light, and magnetism) hydrogels. Furthermore, hydrogels can modify the rheological behavior of fluid systems through their strong consistency and hydrophilicity (Zhang et al., 2022).

The binding of the hydrogel to Pickering particles at the interface increases the resistance of the particles against gastrointestinal enzyme attachment and pH changes and prevents droplet coalescence (Sarkar et al., 2018). PE can be incorporated into the hydrogel network to provide bioactive drugs with better thermal stability and slow release. The Pickering emulsion hydrogel (PEH) was developed as a pH‐sensitive controlled‐release delivery system to address the limitations of Pickering emulsions in some acute or gastrointestinal conditions (Zheng et al., 2022). Since a complex interface is created, it increases the emulsion stability in the gastric environment, and slows down the hydrolysis rate of surfactants. In recent years, emulsion‐based hydrogels have been designed to increase the enzymatic stability of oil‐in‐water emulsions (Lim et al., 2020).

Baneh, or *Pistacia atlantica* subsp. Kurdica, is an endemic plant that belongs to the Anacardiaceae family (Minaiyan et al., 2015; Mirahmadi et al., 2023). Baneh is one of the woody plants in semi‐arid regions of Iran (Esmaili et al., 2022). Its traditional uses, such as anti‐diarrhea, astringent, and improving the symptoms of digestive disorders, are well known (Hetherington & Regan, 2011; Minaiyan et al., 2015). In a previous study, Naji‐Tabasi et al. (2024) designed a novel Pickering emulsion stabilized by 0.1%, 0.3%, 0.5%, and 0.7% (w/w) of Baneh gum (BG) particles. Emulsions stabilized with 0.7% (w/w) BG particles had the highest storage (96.43 ± 0.15%) and centrifuge stability (97.45 ± 1.03%) (Naji‐Tabasi et al., 2024). Curcumin, a lipid‐soluble nutraceutical (Shahbazizadeh et al., 2021), was chosen as a lipophilic encapsulate marker.

In this investigation, we decide to immobilize BG Pickering emulsion droplets in chitosan hydrogel strips using a physical cross‐linking method to develop the stability of the oral delivery system of curcumin with programmed release, which may reveal its potential application, especially in food technology. Chitosan, one of the few natural polymers with a positive charge and biocompatible, non‐toxic, and biodegradable features (Shah, Li, et al., 2016), was selected as the coating layer and hydrogel formation matrix. Chitosan can form a mechanically consistent hydrogel in the presence of trivalent anions, as evidenced by its resistance to gastric digestion (Xiao et al., 2017).

The primary Pickering emulsion, stabilized by 0.7% (w/w) Baneh gum (a natural gum widely available in Iran) and particles (BGPE), was mixed with chitosan solution in different ratios (1:3, 1:5, and 1:7). The adsorbed Baneh gum particles on the oil–water interfaces and the excess non‐adsorbed Baneh gum particles interacted electrostatically with the chitosan matrices to enhance the hydrogel network strength (Lim et al., 2020). A sodium tri‐polyphosphate solution was applied to induce network formation among CS chains. The influence of chitosan concentrations (1.5%, 2.0%, and 2.5% (w/w)) and Pickering emulsion:hydrogel ratios (1:3, 1:5, and 1:7) on the physical properties (storage and thermal antioxidant stability) of emulsion‐filled gel was evaluated. The release of curcumin from Pickering emulsion and emulsion‐filled gel was investigated using in vitro simulated gastric and intestinal fluid.

## 2 MATERIALS AND METHODS

### 2.1 Materials

Baneh gum (BG) was prepared from Baneh forest trees in Kurdistan. Curcumin (purity 95%) was purchased from Sigma Aldrich (USA). Ethanol (96%) was obtained from Khorasan Distillery Company, Iran. Commercial sunflower oil was purchased from the market to prepare emulsions. 2,1 and 2,2‐diphenyl‐1‐picrylhydrazyl (DPPH), bile salt, tripolyphosphate (TPP) salt, and chitosan were purchased from Sigma Aldrich, USA. Sodium azide (NaN_3_) was obtained from Applichem, Germany. Pancreatin enzyme, pepsin, hydrochloric acid, and calcium sodium hydroxide were purchased from Merck, Germany.

### 2.2 Preparation of curcumin‐loaded BG nanoparticles‐stabilized Pickering emulsion

The fabrication of Baneh gum particle suspension was carried out using a method as reported previously by Naji‐Tabasi et al. (2024). For preparation of curcumin‐loaded Baneh gum Pickering emulsion (BGPE), sunflower oil (5.0%) (containing 1.5 mg/mL curcumin) was slowly added to the suspension of solid particles (0.7% w/w) under a homogenizer (Ultra Thorax T25, IKA, Germany) at a speed of 600 rpm. After the addition of oil was complete, homogenization was performed at 16,000 rpm for 2 min to obtain the curcumin‐loaded Baneh gum solid particles‐stabilized Pickering emulsion (cur‐BGPs PE).

### 2.3 Preparation of curcumin‐loaded Pickering emulsion‐filled CS hydrogel

1.5%, 2.0%, and 2.5% (w/w) chitosan (CS) hydrogels were prepared using the ionic gel formation method (Shah, Li, et al., 2016). Briefly, CS solutions were prepared by dissolving pre‐weighted chitosan powder under gentle stirring conditions in a 1.0% (v/v) acetic acid solution. Then, the cur‐loaded BGNPs‐stabilized Pickering emulsion was introduced in the chitosan solutions using a stirrer to mix cur‐BGNPs PE at three different mass ratios of cur‐loaded PE to hydrogel: 1:3, 1:5, and 1:7 (Lim et al., 2020). The mixture was homogenized by an UltraTurrax homogenizer (IKA, Germany) at 8000 rpm for 2 min (Lim et al., 2020). Then, the chitosan solutions containing the cur‐loaded PE were injected into the tri‐polyphosphate salt medium. A sodium tri‐polyphosphate solution at a concentration of 0.1 mg/mL (0.1% w/w) was prepared by dissolving TPP powder in deionized water (pH was adjusted to 4 using acetic acid). The samples were dried in a freeze dryer, and then the stability of curcumin during storage for one month (25°C) was evaluated.

#### 2.3.1 Storage stability of curcumin in Pickering emulsion‐filled hydrogel

The stability of curcumin loaded in the dried Pickering emulsion‐filled CS hydrogel was studied for 21 days of storage (25°C) by measuring curcumin content. To measure the amount of loaded curcumin, the sample dispersion was centrifuged for 40 min at 25°C at 13870 *g*. Then, 0.2 mL of the upper phase was added to the microplate wells. The adsorption rate of samples at 420 nm was read by a spectrophotometer, and the concentration of curcumin was calculated using Equation 1.

(1) $$\text{Concentration}\;\text{of}\;\text{loaded}\;\text{curcumin}\;\%=\frac{\text{Total}\;\mathrm{cur}-\text{free}\;\mathrm{cur}}{\text{Total}\;\mathrm{cur}}\times 100$$

#### 2.3.2 Thermal stability of curcumin in Pickering emulsion‐filled hydrogel

To determine the thermal stability of curcumin in Pickering emulsion‐filled hydrogel, the antioxidant activity of entrapped curcumin in the fresh Pickering emulsion and Pickering emulsion‐filled hydrogel, as well as after thermal treatment (80°C/30 min), was evaluated by the DPPH method, as described by Shah, Li, et al. (2016) and Shah, Zhang, et al. (2016). The absorbance was recorded by a UV–vis spectrophotometer at 517 nm. The antioxidant activity of samples was calculated according to Equation 2 (Hu et al., 2022):

(2) $$\begin{array}{c} \text{DPPH}\;\text{scavenging}\;\text{effect}\;\left(\%\right)\\ =1-\left(\text{sample}\;\text{adsorption}-\text{blank}\;\text{adsorption}\right)/\left(\text{control}\;\text{adsorption}\right)\times 100 \end{array}$$

#### 2.3.3 In vitro release of curcumin in Pickering emulsion‐filled hydrogel

Curcumin released new forms of Pickering emulsion and selected hydrogel (non‐dried) formulations obtained by dialysis at 37°C in the stomach and small intestine condition (Jia et al., 2023; Shah et al., 2021). The dialysis bags were immersed in distilled water for 12 h to remove the preservatives. Then, it was completely washed in distilled water. To examine the stability of the emulsions, the fresh Pickering emulsion (5.0 mL) and hydrogel (2.0 g) were packed into a dialysis bag with a 14 kDa cutoff (Rostami et al., 2023; Shahbazizadeh et al., 2022). Then, it was immersed in 200 mL of gastric juice, under magnetic stirring at 100 rpm and consequently transferred to intestinal release medium on a magnetic stirrer (150 rpm) at a constant temperature (37°C) (Shahbazizadeh et al., 2022). Stomach pH was adjusted to 1.2 using 0.2 M hydrochloric acid and distilled water (500 mL), 1 g sodium chloride, and 0.15 g pepsin (37°C). Intestinal pH was adjusted to 6.8 using 0.1 M sodium, and it contained 0.5 g trypsin in distilled water (500 mL), 3.45 g phosphate buffer (NaH_2_PO_4_), 2.25 g bile salts, and 0.5 mM phosphate pH 6 (37°C). The sample was incubated for 90 min in the simulated stomach environment, and 3.0 mL of the release medium was taken for analysis at time intervals of 0, 30, 60, and 90 min. For the small intestine phase, the sample was kept in simulated small intestine fluid for 120 min. 3 mL of release medium was taken at different time intervals for 0, 30, 60, 90, and 120 min for testing and then replaced with 3 mL of fresh medium. The released curcumin was evaluated by the spectrophotometric method at 420 nm wavelength (Shah, Zhang, et al., 2016).

#### 2.3.4 Assessment of release kinetics

The release kinetics of curcumin from Pickering emulsion and emulsion‐filled chitosan hydrogel were analyzed based on zero‐order (Equation 3), first‐order (Equation 4), Higuchi (Equation 5), and Peppas (Equation 6) models (Shahbazizadeh et al., 2022):

(3) $$M_{0}-M_{t}=k_{0}\cdot t$$

(4) $$\mathrm{Ln}\left(M_{0}/M_{t}\right)=k\cdot t$$

(5) $$Q=K_{H}\cdot t^{1/2}$$

(6) $$M_{t}/M_{\infty }=K_{p}t^{n}$$

where *M*_0_, *M*_*t*_, *t*, and *k* are the initial concentrations of curcumin, the concentration of curcumin in the delivery system at time *t*, and the release constant, respectively. *M*_*t*_/*M*_∞_ is the fraction of curcumin released at time *t*, and *n* is diffusion exponent indicative.

### 2.4 Statistical analysis

A completely randomized design was used for the statistical analysis. The significant difference between the data was determined using ANOVA and Duncan's multiple range tests (*p* ≤ .05). The results were presented as mean ± standard deviation.

## 3 RESULTS AND DISCUSSION

### 3.1 Storage stability of curcumin in BG Pickering emulsion‐filled CS hydrogel

The effect of CS concentration and PE:HYD ratios on the storage stability of curcumin in different formulations is shown in Table 1. The results showed that when the PE:HYD ratios changed from 1:3 to 1:7, and in the same order, in the formulations with the CS concentration above 1.5% (w/w), a more stable emulsion‐filled gel could be formed, which resulted in more survival of curcumin. However, the storage stability of curcumin decreased over time in all treatments. In samples with 1.5% (w/w) CS and a 1:3 EP: HYD ratio, the amount of remaining curcumin reached 62.5% in the 3rd week of storage. Retained curcumin in samples with more than 2.0% (w/w) chitosan and 1:5 and 1:7 EP: HYD ratios at the end of the 1st and 2nd weeks of storage were 100% and 97%, respectively. The highest stability of curcumin after 3rd week of storage was observed in the sample formulated with 2.0% (w/w) CS and a 1:7 EP: HYD ratio (97.36%).

**TABLE 1:** Effect of CS concentration and Pickering emulsion: hydrogel mass ratio on the storage stability of curcumin (%) in chitosan.

CS concentration% (w/w)	PE:HYD	1st week	2nd week	3rd week
1.5	1:3	90.91 ± 1.21^dA^	65.11 ± 2.92^cB^	62.50 ± 2.38^dB^
	1:5	95.31 ± 2.11^cA^	88.47 ± 1.08^dB^	80.61 ± 2.209^cC^
	1:7	100.00 ± 0.00^aA^	90.91 ± 2.88^cB^	81.18 ± 3.01^cC^
2.0	1:3	95.23 ± 0.33^cA^	90.47 ± 3.10^cB^	85.71 ± 3.77^bC^
	1:5	100.00 ± 0.00^aA^	97.29 ± 0.55^bB^	97.14 ± 2.75^aB^
	1:7	100.00 ± 0.00^aA^	97.89 ± 1.73^bB^	97.36 ± 1.50^aB^
2.5	1:3	97.22 ± 1.93^bA^	91.66 ± 1.91^cB^	86.11 ± 2.02^bC^
	1:5	100.00 ± 0.00^aA^	97.96 ± 1.39^bB^	97.29 ± 0.34^aB^
	1:7	100.00 ± 0.00^aA^	100.00 ± 0.00^aA^	96.67 ± 1.03^aB^

*Note*: Different letters indicate significant difference between samples (lowercase letters) and during storage (uppercase letters) (Duncan's test, *p* ≤ .05).

Interestingly, the presence of CS biopolymeric hydrogel and an increase in hydrogel ratio resulted in an increase in cur stability at the end of the storage period due to the increase in covering. The composition of the hydrogel structure reduced the rate of degradation of curcumin in the samples. The storage stability of the entrapped curcumin was significantly dependent on CS concentration and hydrogel mass ratio, and thus the existing strong hydrogen bonding between CS and water molecules enabled it to affect the stability of the CS‐based Pickering emulsion (Pan et al., 2023). In addition, a sufficient concentration of CS can slow the movement of emulsions, form a stable network structure, and promote the stability of curcumin (Liu, Zhang, et al., 2023). The formation of a gel phase affected both the curcumin and the Pickering emulsions (Cavallaro et al., 2019). At concentrations higher than 2.0% (w/w) CS, a more elastic gel‐like structure was created, which can be attributed to the dense network structure formed. A stronger gel structure can preserve curcumin Pickering emulsion during storage. The results showed that high concentrations of CS and high ratios of hydrogel to emulsion could improve the stability of emulsions by inhibiting the aggregation of emulsion droplets (Xu et al., 2022). Therefore, it is inferred that CS hydrogel probably has a reinforced effect on BG Pickering emulsion (Zhang et al., 2015).

By decreasing the CS concentration to less than 2.0%, lower stability (81.18%–62.5%) was observed at the end of the third week. When the CS concentration was low, the Pickering emulsions were unstable against integration. On the contrary, adding more CS made the BG Pickering emulsion more stable. The formation of a gel phase affected the distribution of the oil droplets in the Pickering emulsions (Cavallaro et al., 2019).

Hydrogels must have absolute structural integrity to function as membranes. On the contrary, degradation of the hydrogel can reduce its ability as a physical barrier to protect the curcumin component against oxidants (Heldman et al., 2018). As a result, the hydrogel structure filled with emulsion can reduce the rate of curcumin degradation in the samples. The strengthening effect of cross‐links between anionic polysaccharide (BG) particles and hydrogel network (CS) with positive electric charge increased the stability of the delivery system during storage, followed by promoting the stability of curcumin in the system (Hu et al., 2022). Xia Qiang and Cologuse evaluated the resveratrol (RSV)‐loaded Pickering emulsion‐chitosan hydrogel system. The Pickering emulsion‐filled chitosan hydrogel had excellent stability after 48 days of storage, but the potency of RSV oxidation was unaffected (Wu et al., 2021). According to the results of the 3rd week, samples prepared with 2.0% (w/w) chitosan concentration and a 1:7 emulsion:hydrogel ratio were selected for further studies.

### 3.2 Thermal stability of curcumin in BG Pickering emulsion‐filled CS hydrogel

The antioxidant stability of curcumin after thermal treatment in Pickering emulsion and emulsion‐filled gel is presented in Table 2. After heat treatment, the antioxidant activity of curcumin loaded in Pickering emulsion significantly decreased from 95.21% to 93.60%.

**TABLE 2:** Antioxidant activity of curcumin entrapped in Pickering emulsion and Pickering emulsion‐filled gel after heat treatment.

Sample	DPPH scavenging activity (%) before heat treatment	DPPH scavenging activity (%) after heat treatment
Cur‐loaded BG Pickering emulsion	95.21 ± 0.12^aB^	93.60 ± 0.31^bB^
Cur loaded in BG Pickering emulsion‐filled CS hydrogel	96.84 ± 0.16^aA^	94.40 ± 0.22^bA^

*Note*: Different letters indicate significant difference between samples (uppercase letters) and after heating (lowercase letters) (Duncan's test, *p* < .05).

The TGA results of Baneh gum showed two‐stage decomposition, with the main decomposition of the sample starting above 240–360°C. The first stage occurs at 80°C, which is related to the hydrochloric acid nature of the functional group (Mirahmadi et al., 2023). The heating treatment causes the accumulation of PE and reduces emulsion stability (Xu et al., 2023). However, the antioxidant activity after heat treatment was noticeable. Mirahmadi et al. (2019) reported that Beneh gum had a lower glass transition. In this case, the transition from glass to elastic can easily occur due to water absorption and may cause physical changes such as adhesion (Mirahmadi et al., 2019). This change makes BG Pickering emulsions thermally stable by creating an impermeable layer between the emulsion droplets when heated. The activity of curcumin loaded into BGPE‐filled CS gel against DPPH radicals was higher (*p* ≤ .05) than that of BGPE (94.40%) (Table 2) (Meng et al., 2022). The stability of loaded curcumin mainly depends on the physical stability of the body. When the Pickering emulsion was added to a hydrogel system, it caused the hydrogel to cover the surface of the emulsion droplets as an impermeable coating. The CS hydrogel network that surrounded the curcumin‐loaded BG Pickering emulsion protected these enclosed biomolecules (Paramera et al., 2011).

The addition of CS hydrogel improved the thermal damage of the emulsion model due to higher viscoelasticity and a stronger gel network (Xu et al., 2023). The oil–water interface layer of the droplet became thicker, making it more stable against external stimuli. CS hydrogel maintained its absolute structural integrity to function as a membrane and acted as a second protective layer around curcumin against oxidation initiators. CS and BG biopolymer chains formed complexes through non‐physical interactions, such as ionic bonds, hydrogen bonds, and hydrophobic bonds, and created more advantages, such as more uniform distribution and droplet shape, more emulsion stability, and other physiochemical properties than Pickering emulsion stabilized only by BG particles at various pHs, ionic strengths, temperatures, and longer storage periods (Meng et al., 2022). The presence of hydrophilic groups in the CS structure increased the hydrophilicity of BGPE. The emulsion gel gets less affected by thermally induced flocculation when CS is added to the initial composition. On the other hand, the entanglement of CS with BGPE made a thicker film around the oil droplet interfacial and created high stability against droplet aggregation (Xu et al., 2023) and subsequently provided the conditions for the stability of the loaded curcumin. Furthermore, CS molar mass influenced the glass transition temperature (*T*_*g*_) of composite gel. Probably, the glass transition temperature (*T*_*g*_) of BGPE easily exceeded and led to a soft solid emulsion stabilized by a network of CS (Robin et al., 2021). CS hydrogel has been applied as a vital thermoplastic matrix to form gel composites. The increase in the glass transition of the BGPE‐filled CS gel can be attributed to the polymer chain stiffness and stability due to the addition of the CS hydrogel (Kausar, 2023).

In a similar study, Xu and Academy evaluated the thermal stability of konjac glucomannan (KGM) against gliadin/sodium caseinate nanoparticle Pickering emulsions at 40, 70, and 100°C for 30 min. The addition of konjac glucomannan increases the melting temperature of the Pickering emulsion gel and helps improve the thermal stability of the emulsion gel. The three‐dimensional gel network structure formed by KGM after heating formed a dense interfacial layer on the droplet surface, forming a spatial barrier to prevent the accumulation of oil droplets (Xu et al., 2023). The previous study also reported that curcumin stability against thermal and photochemical treatment significantly improved emulsion gel structure (Liu et al., 2022). Xiong et al. (2016) investigated the antioxidant properties of nanocomposite (NC) hydrogel based on poly(N‐isopropylacrylamide) nanoparticles containing polydopamine. After adding 6 wt% modified poly‐N‐isopropylacrylamide (PDAP) to pNIPAM, the activity of the NC gel is as high as 70%. They stated that nanocomposite gels have higher antioxidant properties (Xiong et al., 2016).

### 3.3 In vitro gastrointestinal release of curcumin in BG Pickering emulsion‐filled CS hydrogel

**FIGURE 1:**
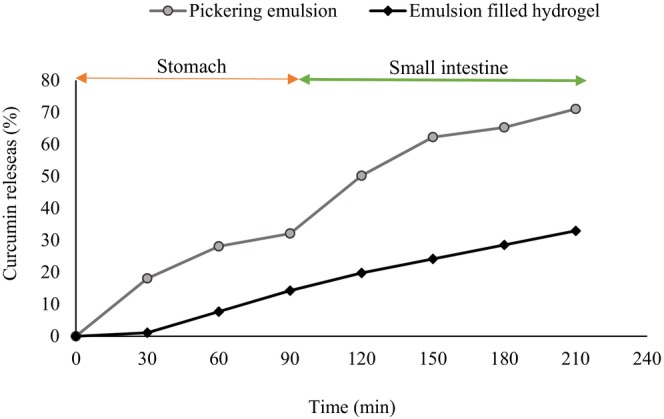
Release of curcumin from Pickering emulsion and emulsion‐filled hydrogel in a simulated gastrointestinal condition.

Figure 1 illustrates the in vitro release profile of curcumin from BGPE and PE‐filled CS hydrogel in simulated gastrointestinal conditions at 37°C using the dialysis bag method.

As can be seen, about 30.0% and 70.0% of curcumin release happened from BGPE after 210 min in both gastric and intestinal fluids, respectively. The protection of curcumin by BGPE was related to its anionic structure in the stomach medium. BG Pickering emulsion shrank when exposed to acidic pH for 90 min, yet it remained intact (Lim et al., 2020). Baneh gum particles, as “anionic polymer”, shrink in acidic environments, which leads to a decrease in the release of bioactive compounds in the stomach environment (Naji‐Tabasi et al., 2017). The slow release of curcumin from anionic polysaccharides in the gastric medium has been confirmed in other studies (Kavousi et al., 2017; Zheng et al., 2022).

As can be seen, about 15.0% and 33.0% of curcumin release happened from BGPE immobilized within CS hydrogel after 210 min in both gastric and intestinal fluid, respectively. By coating the emulsion with CS hydrogel, the amount of curcumin released in the gastric phase was 15% after 90 min of incubation, which indicated increasing the preservation of the integrity of the system in gastric conditions. These results showed that curcumin encapsulated in BG Pickering emulsion‐filled gel is well protected against the effects of stomach enzymes (Hasan et al., 2019). BG Pickering emulsion immobilized within the CS hydrogel matrix exhibited lower curcumin release during SGF digestion. The results can be attributed to the denser structure of the hydrogel, which prevents salt and water from penetrating the gel network and hydrolyzing the CS chains (Liu, Dong, et al., 2023). CS hydrogel swells in an acidic condition, but the creation of hydrogen bonds between the CS hydrogel and BG microgel turned into a stable structure, which controlled water penetration and prevented matrix decomposition. The presence of a binder results in a decrease in the polymer matrix solubility in acidic conditions, which is an effective approach for controlling the release of bioactive compounds. This result was in line with the reports of other researchers (Shah, Li, et al., 2016). Both adsorbed BG particles and unadsorbed BG particles at the oil–water interface electrostatically interact with the CS structure and increase the strength of the hydrogel network (Lim et al., 2020). The electrostatic interaction between anionic BG Pickering particles and CS prevents Pickering emulsion‐filled gel from becoming unstable at an acidic pH (Lim et al., 2020). CS‐coated Pickering emulsion systems are stable in the gastric environment due to steric hindrance and strong positive charge (Jo et al., 2023). Perhaps, the strength of the gel network increased with the addition of CS hydrogel. As a result, the required force to break down the network connections significantly increased. Xu et al. (2019) reported that alginate‐based nanoemulsion‐filled gels improved the bioaccessibility of bioactive compounds (Xu et al., 2019).

Curcumin release was also studied in simulating intestinal fluid. This study was performed after a 90‐minute residence time in the acid secretion, corresponding to the residence time of the emulsion in the stomach. In the simulated intestinal fluid, the release of curcumin from both systems was faster than in the simulated gastric fluid. The intermolecular electrical repulsion inside the network of BG particles increased the basic pH of the intestine, which led to the release of Cur. A similar trend has been reported in other studies (Jelvehgari et al., 2014; Sonia & Sharma, 2011).

As shown in Figure 1, the amount of curcumin released from the BG Pickering emulsion significantly increased (70.0%). In simulated intestinal fluid (SIF) digestion, the BGPE swelled considerably as the free carboxyl groups of BG ionized into carboxylic acid anions by shifting pH from 1.2 to 6.8, and consequently, the continuous release of curcumin happened (Liu, Dong, et al., 2023). Also, the bile salts intensify emulsion instability through mechanisms of emulsifier displacement and replacement at the oil–water interface to prepare the oil droplet to be further digested by enzymes (Ruiz‐Rodriguez et al., 2014). Even though the uncoated Pickering emulsion protected a portion of the droplet surface, the interstices between the BG solid particles could still allow interactions between the oil and bile salts, which could lead to morphological changes, coalescence, and instability of fat during intestinal breakdown (Meng et al., 2022). The CS matrices relatively released the emulsions at near‐neutral pH conditions (Lim et al., 2020).

In simulated intestinal fluid (SIF), curcumin released from CS hydrogel was almost 33.0% at 2 h. The electrostatic interaction between BGPE/CS hydrogels in SIF is weak, which makes the network denser from the hydrogel and leads to the release of curcumin (Liu, Dong, et al., 2023). This result shows that the Pickering emulsion immobilized in the hydrogel exhibits more significant curcumin interaction than the Pickering emulsion alone.

The gel‐like state of the BG Pickering emulsion combined with the strong and dense CS‐hydrogel forms a layer around the droplets, which reduces curcumin release in the intestine by PEHs compared to PE (Meng et al., 2022). In the case of chitosan coatings, these hydrogel layers inhibit the action of bile salts and hinder the breakdown of the emulsions (Meng et al., 2022). BGPE‐filled CS hydrogel prepared by electrostatic interaction resulted in improvement and enhancement of microstructure, viscoelasticity, and self‐supporting (Meng et al., 2022).

This study investigated the effects of pH, sodium chloride, saline, pepsin, and pancreatin on PEHs. Maybe the slow release of curcumin from emulsion‐filled hydrogel in the intestine is related to the absence of human or mouse colonic digestive enzymes such as beta‐glucosidase (Hasan et al., 2019). Another study reported that the release of curcumin from chitosan‐coated liposomes in simulated intestinal fluid was higher than that in simulated gastric fluid, and this was due to cell wall degradation by pancreatic enzymes (Meng et al., 2022).

### 3.4 Curcumin release kinetics

To better understand curcumin release kinetics, the results were analyzed according to mathematical models for drug release, including the zero‐order, first‐order, Higuchi and Peppas zero‐order, and first‐order, Higuchi and Korsmeyer‐Peppas models (Table 3). The correlation coefficient of BG Pickering emulsion obtained for the release kinetics in the small intestine was higher for the Higuchi and Peppas model (*R* ^2^ = .99) compared to the zero‐ and first‐order kinetic models (*R* ^2^ = .85 and .88).

**TABLE 3:** Curcumin release kinetics from Pickering emulsion stabilized with BG particles and BG Pickering emulsion‐filled chitosan gel.

Systems	Medium	Models								
		Zero		First order		Higuchi		Peppas		
		*K*_0_ (%min)	*R* ^2^	*K*_1_ (%min)	*R* ^2^	*K*_*H*_ (%min^1/2^)	*R* ^2^	*K*_*P*_ (%min^*n*^)	*n*	*R* ^2^
BG PE	Stomach	0.00001^a^	.99	0.00032^a^	.91	0.000011^a^	.97	0.0233^a^	0.51^b^	.99
	Intestinal	0.000001^b^	.85	0.00026^a^	.88	0.000012^a^	.98	0.0246^a^	0.50^b^	.99
BG PE‐filled gel	Stomach	0.000001^b^	.90	0.00015^b^	.89	0.000004^b^	.69	–	–	–
	Intestinal	0.000001^b^	.98	0.00017^b^	.98	0.000005^b^	.92	0.0023^b^	0.89^a^	.99

*Note*: Different letters indicate significant difference between samples (Duncan's test, *p* < .05).

The speed of curcumin release (*k*) from the BG Pickering emulsion was higher than that of the emulsion‐filled chitosan gel. The curcumin release from the Pickering emulsion structure from the stomach and intestinal environment was equal to 0.50, which indicates an anomalous transport mechanism. The value of *n* for the release profile of curcumin from emulsion‐filled chitosan hydrogels in the intestinal tract was 0.89, which indicated Case‐II transport (Solghi et al., 2020). These results are consistent with previous studies showing curcumin release from CS scaffolds.

## 4 CONCLUSION

The main purpose of the emulsion‐filled hydrogel system is to encapsulate bioactive compounds to protect them from the severe impact of the stomach environment, and the present study achieved a favorable result. It can be concluded that after exposure to gastric fluid, BG Pickering emulsion‐filled CS hydrogel had a more controlled release and overcame the limitations of Pickering emulsions in harsh gastrointestinal conditions and thermal treatment. This effect is probably related to the higher protective effect of the hydrogel polymer matrix. It was also found that higher concentrations of chitosan and higher mass ratios of hydrogel:emulsion showed greater stability. The formation of the electrostatic complex between BG microparticles and CS hydrogel significantly increased the stability and antioxidant activity of curcumin. Incorporating Baneh gum particles into CS hydrogels improved the delivery performance and provided a novel design for the stability and pH‐responsive release of hydrophobic compounds in food applications.

## AUTHOR CONTRIBUTIONS

**Sara Naji‐Tabasi:** Data curation (equal); funding acquisition (equal); investigation (equal); supervision (equal); validation (equal); writing – review and editing (equal). **Monir‐sadat Shakeri:** Data curation (equal); project administration (equal); writing – review and editing (equal). **Atena Modiri‐Dovom:** Investigation (equal); methodology (equal). **Saeedeh Shahbazizadeh:** Writing – review and editing (equal).

## CONFLICT OF INTEREST STATEMENT

The authors declared that there is no competing interest.

## ETHICAL APPROVAL

This article does not contain any studies with human participants or animals performed by any of the authors.

## CONSENT FOR PUBLICATION

The authors hereby consent to the publication of the work.

## Data Availability

Data are available on request from the authors.
